# Knowledge Gaps and Bridges: The Relationship between the Awareness of General Patient Rights and the Awareness of Minors’ Patient Rights in the Netherlands

**DOI:** 10.3390/children11010109

**Published:** 2024-01-16

**Authors:** Charelity Adu-Gallant, Jaan Toelen, Judith Sluiter-Post, David De Coninck, Peter de Winter

**Affiliations:** 1 Pediatric Department, Spaarne Gasthuis, 2035 RC Haarlem and 2134 TM Hoofddorp, The Netherlands; 2 Pediatric Department, OLVG, 1091 AC Amsterdam, The Netherlands; 3 Department of Development and Regeneration, KU Leuven, 3000 Leuven, Belgium; 4 Child and Youth Institute Leuven, KU Leuven, 3000 Leuven, Belgium; 5 Pediatric Department, UZ Leuven, 3000 Leuven, Belgium; 6 Spaarne Gasthuis Academy, Spaarne Gasthuis, 2134 TM Hoofddorp, The Netherlands; 7 Centre for Sociological Research, KU Leuven, 3000 Leuven, Belgium

**Keywords:** patient rights, minors, age-based framework, medical decision-making, consent, self-determination, autonomy, majority, Medical Treatment Agreement Act (WGBO)

## Abstract

The major focus of this research is the level of awareness among Dutch parents of general patient rights in relation to minors’ patient rights. Moreover, this study is intended to highlight the most effective strategies to increase the awareness of general and minor patient rights in the Netherlands. A survey was conducted among 1010 Dutch parents aged between 35 and 55 years who had at least one child. In this study, we described the relationship between the knowledge among parents of general patient rights and their understanding of the patient rights of minors. A significant connection was found between the knowledge levels of general patient rights and the knowledge levels of the patient rights of minors (*p* < 0.05 [95% CI: 0.019–0.183]). While age and sex (male/female) did not appear to be significant confounders in this association, the educational background of the participants may have played a role. This study provides comprehensive insights into the association between the knowledge of general patient rights and the patient rights of minors among Dutch parents. Furthermore, this study points out that there is a need for focused educational interventions to address specific areas of misunderstanding or uncertainty.

## 1. Introduction

In the Netherlands, patient rights are established in several laws, such as the Medical Treatment Agreement Act (WGBO) [1,2], the Care and Coercion Act (Wzd) [3,4], and the Healthcare Quality, Complaints and Disputes Act (Wkkgz) [5]. The Dutch Medical Treatment Agreement Act (WGBO) lays the foundation for safeguarding patient rights in the Netherlands, and it was enacted to ensure a transparent and patient-centered healthcare system [6,7].

Under the WGBO, patients have the right to be fully informed about their medical condition, diagnosis, and available treatment options [1,2,6,7]. Healthcare providers must provide clear and understandable information, allowing patients to make informed choices regarding their health [8]. Moreover, patients have the right to give or withhold consent for any medical procedure, treatment, or examination [1,2,6,7,8]. Informed consent is a fundamental aspect of the doctor–patient relationship, emphasizing the importance of respecting a patient’s autonomy and individuality [8]. The right to free choice in Dutch healthcare means that patients can choose their healthcare provider, including their doctors, hospitals, and specialists. This principle aims to give patients more control over their healthcare decisions. Dutch citizens are generally allowed to select their preferred healthcare provider within certain regulations and guidelines set by the Dutch healthcare system [1,8].

Additionally, the Medical Treatment Agreement Act (WGBO) guarantees patients access to their medical records, allowing them to review their medical history, diagnoses, and treatments [1,9]. This transparency promotes a sense of trust between patients and healthcare providers. Moreover, patients have the right to request a second opinion from another healthcare professional [1]. This ensures that patients can explore alternative treatment options and confirm the initial diagnosis or recommended course of action [1,3,10].

The Medical Treatment Agreement Act (WGBO) also includes the patient rights of minors [11] in the Netherlands [1,12]. The legal position of minors (people under the age of 18 years) and the age at which minors are allowed to make medical decisions differ in European countries [13,14]. Let us summarize. Two frameworks cover minors’ legal position in healthcare: the “age”-based framework and the “maturity”-based framework [15,16]. In the Netherlands, medical decision-making without parental permission is allowed from the age of 16 [1,12,16].

Despite the legal framework provided by the Medical Treatment Agreement Act (WGBO) and the other acts, it is essential to acknowledge the role of patient awareness in upholding these rights. Patient rights are fundamental principles safeguarding the autonomy, dignity, and well-being of individuals seeking healthcare services [17,18,19]. Understanding and being aware of these rights is essential for patients to actively participate in their medical care decisions and ensure that they receive the best possible treatment and care [17,18,19].

Worldwide, several studies have concluded that most patients are not sufficiently aware of their patient rights [19,20,21,22,23,24]. In 1998, two-thirds of Dutch citizens had never heard of the patient rights and obligations established in the Medical Treatment Agreement Act (WGBO) [25]. In 2006, the results of a survey about the Medical Treatment Agreement Act (WGBO) showed that one-third of the patients did not know about the different patient rights and obligations [26].

In recent years, increasing attention has been given to educating patients about their rights to promote a more patient-centered healthcare system. Currently, information about the Medical Treatment Agreement Act (WGBO) is available online. There are multiple online articles about Dutch patient rights [27,28,29,30,31]. Several institutions, such as “The Dutch Patient Federation” [32] and “The Ombudsman for Children” [33], play an informative and advisory role vis-à-vis patients.

Our recent study about parental knowledge of the patient rights of minors showed that only a minority of participating parents knew that their children were legally allowed to make medical decisions (without parental consent) from age 16 [16]. However, no recent studies have examined parents’ awareness of general patient rights. What do parents know about their patient rights? And how does this correlate with the knowledge of minors’ patient rights?

Therefore, the aim of this study was to explore the parental understanding of the rights of minors based on parents’ knowledge of general patient rights. It was hypothesized that having a better understanding of general patient rights could contribute to a better understanding of patient rights for minors.

## 2. Materials and Methods

### 2.1. Study Design

This study was performed in the Netherlands. Survey-based research was conducted to answer the research questions.

### 2.2. Data Collection

Data were collected via a European research agency (Bilendi & Respondi). Parents affiliated with this research agency receive emails with questions to participate in online surveys. As a reward for completing surveys, parents receive loyalty points with which they can buy items of their choice. The survey was sent to all eligible households, and 1010 Dutch parents between the ages of 35 and 55 who had at least one child completed the survey. Inclusion stopped when the total number of 1010 parents was reached. Parents received an informed consent form before accessing the survey. Access to the survey was denied when parents disagreed with the informed consent form. The survey ran from 23 May 2022 to 10 June 2022 with a response rate of 32%.

### 2.3. Survey and Assessment

The survey consisted of various questions and statements about patient rights. Experienced disciplinary judges who were specialized in healthcare law evaluated and completed the questionnaire. The answers of the parents were compared with the answers of the disciplinary judges.

The survey was divided into several parts. The first part consisted of multiple-choice questions about general patient rights. This part of the survey consisted of twenty statements about general patient rights. The questions had three response options (applicable/not applicable/do not know). The option “I don’t know” was added to distinguish between conscious incapability and unconscious incapability.

The multiple-choice questions covered different topics, namely:

1. The right to information (the Medical Treatment Agreement Act (WGBO)) [1,2,6,7,8]
2. The right to consent (the Medical Treatment Agreement Act (WGBO)) [1,2,6,7,8]
3. The right to free choice (the Healthcare Insurance Act (ZVW)) [34]
4. (Legal) representation (the Medical Treatment Agreement Act (WGBO)) [1,2]
5. Medical records (the Medical Treatment Agreement Act (WGBO)) [1,2,9]
6. The right to privacy and confidentiality (the Medical Treatment Agreement Act (WGBO)) [1,2]
7. The right to self-determination/autonomy (the Medical Treatment Agreement Act (WGBO)) [1,2]

The second part of the survey consisted of questions about parents’ experiences and preferences regarding information provision.

### 2.4. Statistics

Statistical analysis was performed using IBM’s SPSS Statistics, version 25.0. Percentages were calculated to determine how much Dutch parents knew about general patient rights. Linear regression analysis was performed to identify a correlation between the dependent variable (the knowledge of minor patient rights) and the independent variable (knowledge of general patient rights) and to identify possible confounders.

## 3. Results

### 3.1. Characteristics of the Participants

Data from 1010 surveys were collected, and 65.7% (N = 664/1010) of the participating parents were female. The mean and median ages of the parents were 45 years (age range: 35 to 55), and 41.2% (N = 416/1010) of the parents lived in the central region of the Netherlands. Furthermore, 44.3% (N = 447/1010) of the parents had obtained a higher vocational education (HBO) or university degree. Sociodemographic characteristics are shown in Table 1. Only seven parents explained that the Netherlands was not their country of birth.

### 3.2. Parents’ Knowledge of General Patient Rights

In total, 50% of the participating parents answered 80–100% of the questions correctly. This was equal to 16 to 20 correct responses to statements. See Table 2.

The survey results indicate that 87.4% of the parents were aware of their right to information, and 44.5% of the parents were aware of the right not to know information about their health condition. Additionally, most of the parents were aware of their right to consent freely to a treatment or diagnostic examination or to refuse treatment, at 84.6%, 80.1%, and 78.1%, respectively. However, only 66.1% of the parents knew that, in acute life-threatening situations, a doctor could make a decision instead of the patient. Concerning free choice, the results show that 87% of the parents were aware of the right to choose a health insurance policy. Over 81% of the parents knew they were free to choose a general practitioner, and over 70% of the parents knew they were free to choose a hospital. Solely 40% of the parents knew that, as a patient, you have the right to choose which hospital you want to be taken to by ambulance. Regarding legal representation, over 75% of the parents knew they had the right to be represented when they could not exercise their rights themselves. The results are shown in Table 3.

Furthermore, the results show that most of the parents (85.7%) knew that the involved healthcare providers must maintain their medical patient files. Additionally, over 83% of the parents were aware of their right as patients to inspect and copy their own patient files. Concerning the right to privacy and confidentiality, 85.6% of the parents were aware that their privacy must be protected. Nearly 80% of the parents also knew that it was their choice to have additional, unnecessary medical care or people present during a medical examination or treatment if such a presence was not necessary.

Lastly, the parents were administered questions about the right to self-determination/autonomy. The results show that over 79% of the parents knew that they had the right to a dignified end of life, and 75.3% of the parents were aware of the right to request an abortion for medical reasons. Concerning abortion for psychological reasons, just 58.9% of the parents were aware of this right. Remarkably, 42.4% of the parents thought they had the right to euthanasia by a doctor even if the doctor disagreed with this. Moreover, 43.2% of the parents also thought they had the right to demand/receive treatment if they felt it was the best treatment for them, regardless of the doctor’s judgment.

### 3.3. The Relationship between the Knowledge of General Patient Rights and the Knowledge of Minor Patient Rights

A significant association was found between the knowledge of general patient rights among parents and their understanding of minor patient rights (*p* < 0.05 [95% CI: 0.019–0.183]). For every one-unit increase in the parental knowledge of general patient rights, a 0.101-unit increase in the knowledge of minor patient rights was predicted (Beta = 0.077, t = 2.422, *p* = 0.016). The t-value associated with parental knowledge was 2.422, and the *p*-value was 0.016, both of which were below the conventional significance threshold of 0.05. This suggests that the relationship is unlikely to have occurred by chance. The 95% confidence interval for the coefficient (0.019 to 0.183) further supports the significance of this association.

Age had a coefficient of 0.098 (Beta = 0.020, t = 0.632, *p* = 0.527), indicating a weak positive association with parental knowledge of minor patient rights. However, this result was not statistically significant (*p* = 0.527), suggesting that age may not be a confounding factor in the relationship between parental knowledge of general patient rights and parents’ knowledge of minor patient rights. The coefficient for sex (male/female) was 0.731 with a Beta of 0.012, and this result was not statistically significant (*p* = 0.707). This indicates that the sex of parents may not be a confounding variable in the relationship between parental knowledge of general patient rights and parents’ knowledge of minor patient rights. The wide confidence interval (−3.080 to 4.541) also suggests a lack of precision in estimating the effect of sex.

Parents with a higher-level degree tended to have more knowledge of minors’ patient rights, as indicated by the coefficient of 3.764 (Beta = 0.064, t = 2.008, *p* = 0.045). This result was statistically significant, suggesting that education level could be a confounding factor in the relationship between parental knowledge and parents’ knowledge of minor patient rights.

The results are shown in Table 4.

### 3.4. Information Provision

**Figure 1:**
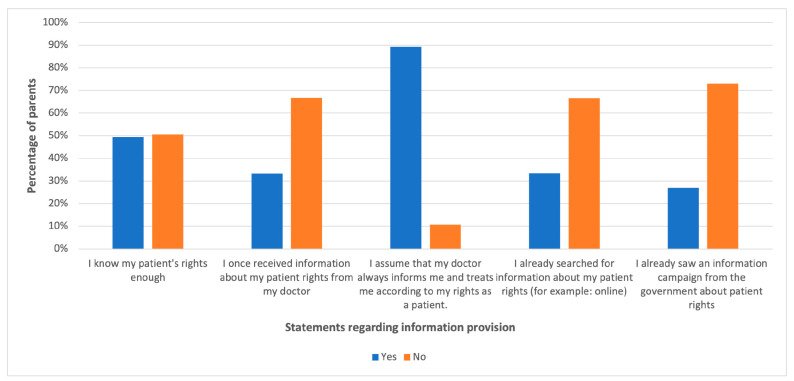
Information provision.

Regarding information provision, 49.4% of the participating parents answered that they had sufficient knowledge about their patient rights. In total, 902 (89.3%) parents assumed that their doctor always informed and treated them according to their patient rights. See Figure 1.

**Figure 2:**
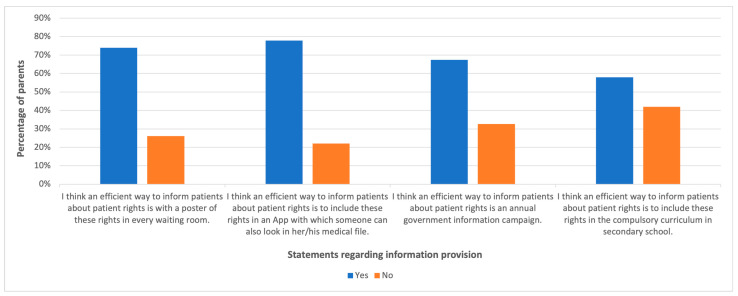
Information provision.

As shown in Figure 2, the most favorable way (77.9%) to inform patients about their patient rights is to include the patient rights in an app with which someone can also look at her/his medical file. The less favorable way (58%) to get informed about patient rights, according to parents, is to have patient rights in the compulsory curriculum in secondary school.

## 4. Discussion

### 4.1. Parental Knowledge of General Patient Rights

The findings of this study suggest that the majority of the participating parents were aware of most of the general patient rights concerning the right to information, the right to consent, the right to free choice, the right to legal representation, the right to access medical records, and the right to privacy and autonomy. In total, 50% of the parents answered 16 to 20 questions correctly. This level of awareness among Dutch parents is notably positive, indicating a foundational understanding of patient rights. Worldwide, several studies have examined the awareness of patient rights in their region or country. Our findings are consistent with studies from Saudi Arabia [17] and Poland [18], where the overall awareness of patient rights was adequate. However, our findings contradict multiple studies whose results have suggested that patients were not sufficiently aware of their patient rights [19,22,23].

### 4.2. Parental Knowledge of the Patient Rights of Minors Based on Parents’ Knowledge of General Patient Rights

Many principles of patient rights are common across age groups, such as the right to information, informed consent, confidentiality, and privacy. While general patient rights provide a baseline, there are specific considerations and nuances when it comes to minors. These may include the role of parental consent, the evolving capacity of minors, and the balance between respecting autonomy and ensuring the best interests of the minor.

In the context of Dutch healthcare, self-determination or autonomy is a crucial aspect of patient rights, even for minors. However, the autonomy of minors is often subject to some limitations and considerations. In the Netherlands, the legal framework acknowledges the capacity of minors to make decisions about their healthcare. As a general rule, the principle is that minors under the age of 12 are presumed to lack the capacity to make independent medical decisions. Minors between 12 and 16 years old are increasingly being recognized for their autonomy, and they are typically involved in decision-making processes regarding their treatment, taking into account their level of maturity. From the age of 16, minors are allowed to consent to medical diagnostics and treatments without parental consent. A previous study about the patient rights of minors showed the importance of improving the knowledge of parents on the patient rights of minors [16]. It showed that parents are not sufficiently aware of the age-based framework that applies to minors in Dutch healthcare [16]. Moreover, the parental knowledge of the age-based framework varied, depending on the topic [16]. Regarding euthanasia and children’s rights in the Netherlands, Dutch law allows euthanasia for minors under certain conditions. A child between the ages of 12 and 16 must have parental consent, and children aged 16 and 17 must involve their parents in the decision-making process, but they can ultimately decide for themselves.

Self-determination and autonomy are fundamental principles within Dutch healthcare, aiming to empower patients to actively participate in their medical decisions and prioritize their individual preferences and values. To make an informed choice, it is necessary to be well-informed about a subject. Regarding self-determination and autonomy, a lack of knowledge about these patient rights can lead to conflicts and disagreements between patients and healthcare professionals.

In this study, we assessed parents’ understanding of the rights of minors based on their knowledge of general patient rights. The results show a noticeable trend in which an increase in the knowledge of general patient rights led to a slight rise in understanding minors’ rights. This supports the hypothesis that better knowledge of general patient rights is associated with an improved understanding of minors’ patient rights. As mentioned before, many principles of patient rights are common across age groups. The more you know about general patient rights, the more you know about the patient rights of minors. Knowledge of general patient rights provides a baseline on which you can build knowledge of the patient rights of minors.

The study’s findings suggest that the highest degree attained by parents is a statistically significant predictor of the knowledge of minors’ patient rights. While the effect size was relatively small, the significance of the result supports the idea that the association between the knowledge of general patient rights and minors’ patient rights may be partially explained by the educational background of parents. The association between educational level and the awareness of patient rights has also been documented by Kagoya et al. [20], Zülfikar and Ulusoy [22], and Agrawal et al. [24], but it contradicts the findings of Aljeezan et al. [17] and Unnikrishnan et al. [21]. The results of Aljeezan et al. [17] and Unnikrishnan et al. [21] suggested no association between educational level and the awareness of patient rights. Researchers may further investigate the association between educational background and awareness of patient rights and the specific mechanisms through which education influences the awareness of the patient rights of minors to gain a more nuanced understanding of this relationship.

Age and sex do not appear to be significant confounders in the association between parental knowledge of general patient rights and their knowledge of the patient rights of minors. Concerning age, these findings are consistent with the study by Madadin et al. [19]. These researchers stated that there is no association between age and the awareness of patient rights. However, different reports have shown a relationship between age and the level of awareness of patient rights. While Aljeezan et al. [17] stated that an increase in age is associated with an increase in knowledge of patient rights, the findings of Agrawal et al. [24] suggest that younger adults are more aware of patient rights than older adults. According to Madadin et al. [19], the discrepancy between different studies could be due to differences in mean ages. Concerning sex, it is difficult to compare our study to other studies because, often, the word “gender” is used to describe “sex”. When it comes to gender, some studies have reported an association with knowledge of patient rights [24], and some reports have not suggested this association [17]. Despite the lack of significance in this recent study, it is essential to consider the possibility that age and sex might still be confounders, though not captured by this analysis. Additionally, exploring other potential confounding variables not included in the current study may further enhance this study’s findings.

### 4.3. Information Provision

This study shows that various methods can be used effectively to educate patients about their rights. Among the findings, the majority of parents were found to prefer having an app where patient rights are documented and which also gives access to their medical records, indicating a desire for simple and easy-to-understand information. In addition, showing patient rights on posters in waiting areas or in regular, government-driven awareness campaigns has been indicated as another effective method. Conversely, the least preferred way was incorporating patient rights into compulsory school curricula, probably because of the general aversion to compulsion and the challenge of attracting minors towards such subjects. This indicates that there is a need to implement new communication strategies for reaching out to diverse audiences.

The study indicates that fewer than 30% of the participating parents had ever come across any government-supported campaign about patient rights. This result is significant, especially when almost 90% of the parents stated that their doctors always notified them about these rights and observed them, while just below one-third (fewer than 35%) had ever received such information from their healthcare providers. This mismatch reveals a significant problem in informed consent practice, which is essential in enabling patients to make informed choices regarding their healthcare. Informed consent constitutes an essential part of patient rights since it ensures that patients are fully aware of everything they might have to deal with before making any decision concerning healthcare. As such, healthcare givers must ensure that they treat their patients as per the prescribed codes while providing them with ample education on these codes’ provisions. The latter part is crucial in helping patients become active participants in healthcare so as to receive the best possible treatment.

In terms of information availability, fewer than 30% of the participating parents had encountered government-sponsored efforts aimed at informing people about patient rights. However, despite almost all parents thinking that doctors continuously communicated with them about these laws and abided by them as well, only less than thirty-five percent had ever received such information from healthcare providers. This shows a fundamental flaw in informed consent, which is the basis for patients to make informed choices about their health. For a patient to take part in his or her own health decision-making processes, informed consent must be available. Therefore, healthcare providers should not only comply with patient rights but also enlighten them on these rights themselves. The need for this is strong because it empowers patients to be active players in their own therapy, as well as getting the best services in that regard. Based on our findings, we think it is essential to take a multi-faceted approach when it comes to educational interventions. First, we need a government-led information campaign that focuses on patient rights. This should expand to schools and healthcare facilities where information can be passed quickly.

### 4.4. Strengths and Limitations

There were several strengths in this survey-based study. First, it applied a cost-effective and flexible research methodology. Second, it filled a gap in the related literature by examining a general understanding of patient rights as well as minors’ rights in the Netherlands that had not been investigated recently. Thirdly, the study was conducted with 1010 participants, and all data were available; hence, there was no need to exclude any survey from the statistical analysis. It represented various parts of the Netherlands by considering different places and reflecting national educational distribution. Fourthly, a linear regression analysis was performed, taking various confounders into account.

However, there were a number of limitations to the present research. For example, it was only natural that some respondents who were affiliated with an agency and reimbursed for taking part in surveys may not have given their truthful responses at all times; this might constitute a limitation of this study. Also, another limitation was that some respondents could have sought information from internet sources while answering questions online. To counter this, the research company omitted questionnaires that were completed too quickly or very slowly. The response rate in our study was 32%, and this value is actually pretty standard for a large questionnaire study involving the general public. Furthermore, we did not specifically ask if both of a child’s parents were that child’s legal guardians. Since knowing whether both parents were married or not would not accomplish anything when it comes to understanding why people were not aware of their patients’ rights, we decided not to include this information in our analysis.

However, these measures did not rule out potential selection bias since parents who required more time or wanted to fill out the survey later may have been less likely to respond. We acknowledge the significance of providing a demographic breakdown in our survey. This insight is invaluable since immigrants may possess varying levels of knowledge in specific areas. We checked our database, and only seven parents explained that the Netherlands was not their country of birth. In this study, we focused on parents of Dutch origin. We agree that it is fascinating to question immigrants and refugees on this healthcare issue.

Finally, the findings cannot be generalized since only parents aged between 35 and 55 years who had at least one child participated in this study. Moreover, ethnicity and native language variations, which could act as considerable correlation factors, were not taken into account as far as possible divergent responses were concerned in this study.

## 5. Conclusions

In conclusion, this research aimed to explore the parental understanding of the rights of minors based on parents’ knowledge of general patient rights. The results suggest that an increase in the knowledge of general patient rights leads to a slight rise in the understanding of minors’ patient rights. The educational background of a parent is a statistically significant predictor of the awareness of minors’ patient rights.

The high levels of awareness in many areas of general patient rights signal progress in disseminating information about general patient rights. However, this study also points out that there is a need for focused educational interventions to address specific areas of misunderstanding or uncertainty.

Our findings are crucial since they signify common ground between knowledge about general patient rights and knowledge about the patient rights of young people. Consequently, these connections have implications for new policies and teaching strategies that will advocate for a more targeted approach to raising awareness of patient rights. In particular, this approach should focus on the unique rights of minors. Therefore, healthcare facilities have to work closely with government institutions in order to provide orientation on the rights of such patients as children or teenagers, who are often ignorant about them. That is when patients can make informed decisions and healthcare systems can be centered upon care recipients.

This study has far-reaching implications for future studies since it opens up more research avenues. It is imperative to further investigate how knowledgeable minors are regarding their own rights and which roles physicians, health professionals, and parents may play in enhancing such awareness. Consequently, this is more than just research, and we hope it will serve as an eye-opener into better healthcare in the future.

## Figures and Tables

**Table 1:** Sociodemographic characteristics of the participating parents.

		Total (N = 1010)
Sex (%)	Male	346 (34.3%)
	Female	664 (65.7%)
Age (years, mean (range))		45 (35–55)
Highest degree (%)	No further education	167 (16.5%)
	Secondary vocational education (MBO)	396 (39.2%)
	Higher vocational education (HBO)/university	447 (44.3%)
Born in the Netherlands	Yes	1003 (99.3%)
	No	7 (0.7%)

**Table 2:** Parental knowledge of general patient rights.

Level of Knowledge (%)	Percentage of Parents
Poor knowledge (answered 0–50% of the questions right)	13.9% (N = 140)
Average knowledge (answered 50–80% of the questions right)	36.1% (N = 365)
Good knowledge (answered 80–100% of the questions right)	50.0% (N = 505)
Total	100% (N = 1010)

**Table 3:** Statements about general patient rights and the responses of the parents results of the survey about general patient rights. Overview of the statements, divided into different topics.

Topic	Statement	Correct	Incorrect	“I Don’t Know”
The right to information	The patient has the right to information about her/his state of health.	87.4% (N = 883)	7.6% (N = 77)	5.0% (N = 50)
	The patient has the right not to know information about her/his health condition.	44.5% (N = 449)	35.7% (N = 361)	19.8% (N = 200)
The right to consent	The patient can freely consent to a treatment.	84.6% (N = 854)	7.2% (N = 73)	8.2% (N = 83)
	The patient can freely consent to a diagnostic examination.	80.1% (N = 809)	8.9% (N = 90)	11.0% (N = 111)
	The patient has the right to refuse well-defined, and even lifesaving, treatment.	78.1% (N = 789)	9.5% (N = 96)	12.4% (N = 125)
	In acute life-threatening situations, the doctor can decide instead of the patient.	66.1% (N = 668)	15.9% (N = 161)	17.9% (N = 181)
The right to free choice	The patient is free to choose her/his health insurance policy.	87.0% (N = 879)	6.7% (N = 68)	6.2% (N = 63)
	The patient is free to choose her/his general practitioner.	81.5% (N = 823)	11.8% (N = 119)	6.7% (N = 68)
	The patient is free to choose her/his hospital.	70.2% (N = 709)	18.7% (N = 189)	11.1% (N = 112)
	The patient has the right to choose which hospital the ambulance takes her/him to, regardless of distance/circumstances.	39.8% (N = 402)	42.4% (N = 428)	17.8% (N = 180)
(Legal) representation	The patient is represented if she/he is unable to exercise his/her rights.	75.3% (N = 761)	9.7% (N = 98)	15.0% (N = 151)
Medical records	The patient can count on a carefully kept patient file.	85.7% (N = 866)	7.5% (N = 76)	6.7% (N = 68)
	The patient has a direct right to inspect and receive a copy of her/his patient file.	83.2% (N = 840)	9.4% (N = 95)	7.4% (N = 75)
The right to privacy and confidentiality	The patient is assured of the protection of her/his privacy.	85.6% (N = 865)	7.8% (N =79)	6.5% (N = 66)
	The patient may object to the presence of persons during a medical examination or medical treatment when such a presence is not strictly necessary for medical care.	79.7% (N = 805)	8.8% (N = 88)	11.5% (N = 116)
The right to self-determination/autonomy	The patient has the right to a dignified end of life.	79.1% (N = 799)	8.9% (N = 90)	12.0% (N = 121)
	A pregnant woman can request an abortion for medical reasons.	75.3% (N = 761)	9.1% (N = 92)	15.5% (N = 157)
	A pregnant woman can request an abortion for psychological reasons.	58.9% (N = 595)	13.5% (N = 136)	27.6% (N = 279)
	The patient has the right to euthanasia by a doctor, even if the doctor does not agree to this.	35.3% (N = 357)	42.4% (N = 428)	22.3% (N = 225)
	The patient has the right to demand/receive treatment if she/he thinks it is the best treatment for her/him, regardless of the doctor’s judgment.	34.4% (N = 347)	43.2% (N = 436)	22.5% (N = 227)

**Table 4:** The association between the knowledge of general patient rights and the knowledge of minor patient rights.

Model		Unstandardized Coefficients ^1^		Standardized Coefficients ^2^	t-Statistic ^3^	*p*-Value ^4^	95% Confidence Interval ^5^	
		B	Standard Error	Beta (β)			Lower Bound	Upper Bound
1	Parental knowledge of the patient rights of minors (constant) ^a^	5.618	8.002		0.702	0.483	−10.086	21.321
	Parental knowledge of general patient rights ^b^	0.101	0.042	0.077	2.422	0.016 *	0.019	0.183
	Age ^c^	0.098	0.155	0.020	0.632	0.527	−0.206	0.402
	Sex (male/female) ^c^	0.731	1.942	0.012	0.376	0.707	−3.080	4.541
	Highest degree ^c. d^	3.764	1.875	0.064	2.008	0.045 *	0.085	7.444

Linear regression analysis. ^a^ Dependent variable: parental knowledge of the patient rights of minors. ^b^ Independent variable: parental knowledge of general patient rights. ^c^ Predictors: age, sex (male/female), and highest degree. ^d^ Highest degree: no further education and secondary vocational education (MBO), coded as 0. Higher vocational education (HBO)/university, coded as 1. ^1^ B (unstandardized): the change in the dependent variable for a one-unit change in the independent variable while holding the other variables constant. Its value applies the unit of the dependent variable. Standard error: a measure of how much the estimated coefficient was expected to vary from the true population coefficient. ^2^ Beta-coefficient: the change in the dependent variable in terms of standard deviation for a one-standard-deviation change in the independent variable. ^3^ T-statistic: a measure of how many standard errors the coefficient estimate was from zero. It was calculated by dividing the estimated coefficient by its standard error. A higher absolute value of the T-statistic indicates that the variable is likely to be more important, and if the T-statistic is associated with a low *p*-value, it suggests that the variable is statistically significant in predicting the dependent variable. ^4^ *p*-value: the probability of obtaining the observed results if the null hypothesis (no effect) were true. *p* < 0.05 * indicates that the variable is statistically significant, suggesting that the relationship between the dependent variable and independent variable is likely not due to random chance. ^5^ 95% confidence interval: we are 95% confident that the true value of the coefficient falls within this range.

## Data Availability

The data presented in this study are available on request from the corresponding author. The data are not publicly available due to the ethic and privacy.
